# A Novel PKD1 Mutation Associated With Autosomal Dominant Kidney Disease and Cerebral Cavernous Malformation

**DOI:** 10.3389/fneur.2018.00383

**Published:** 2018-05-25

**Authors:** Christian Thomas, Andrea Zühlsdorf, Konstanze Hörtnagel, Lejla Mulahasanovic, Oliver M. Grauer, Philipp Kümpers, Heinz Wiendl, Sven G. Meuth

**Affiliations:** ^1^Clinic of Neurology with Institute of Translational Neurology, University of Münster, Münster, Germany; ^2^Department of General Pediatrics, Metabolic Diseases, University Children's Hospital Münster, Münster, Germany; ^3^CeGaT GmbH and Praxis für Humangenetik Tübingen, Tübingen, Germany; ^4^Division of General Internal Medicine, Nephrology, and Rheumatology, Department of Medicine D, University Hospital Münster, Münster, Germany

**Keywords:** ADPKD, CCM, sequencing, familial, mutation

## Abstract

Autosomal dominant polycystic kidney disease (ADPKD) is a genetic disorder characterized by the presence of renal cysts and specific extrarenal abnormalities. ADPKD is caused by mutations in either *PKD1* or *PKD2* genes that encode for integral membrane proteins Polycystin-1 (PC1) and Polycystin-2 (PC2), respectively. Extrarenal involvement includes noncystic manifestations such as dilatation of the aortic root, artery dissection and intracranial aneurysms. Cerebral cavernous malformation (CCM) is a rare vascular malformation disorder characterized by closely clustered and irregularly dilated capillaries that can be asymptomatic or cause variable neurological manifestations, such as seizures, non-specific headaches, progressive or transient focal neurologic deficits, and cerebral hemorrhages. Familial CCM is typically associated with mutations in *KRIT1* (*CCM1*), *CCM2*, and *PDCD10* (*CCM3*). The co-occurrence of ADPKD and CCM has been previously described in a single patient, although genetic analysis was not performed in this study. We report here a family with ADPKD associated with CCM in two sisters. Direct sequencing of the index patient revealed a single novel heterozygous frameshift mutation in *PKD1*, and lack of mutations in genes usually related to CCM. This suggests that CCM represents an additional phenotype of ADPKD.

## Introduction

**Figure 1:**
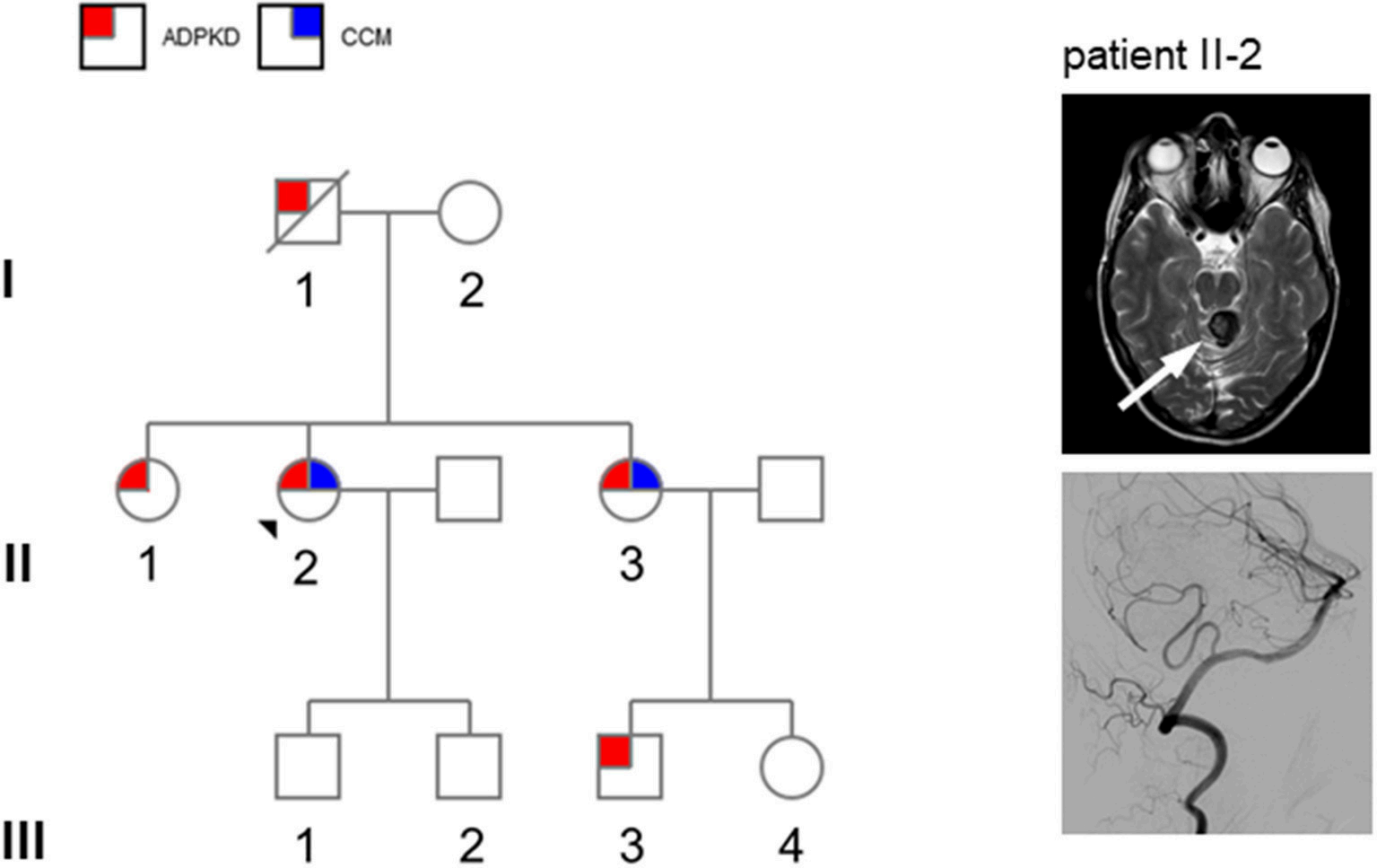
**Left**: pedigree of the present family (patient II-2: propositus). Circles denote females and squares males. Red squares denote autosomal dominant kidney disease (ADPKD) and blue squares denote cerebral cavernous malformations (CCM). **Right**: MRI showing presence of cerebral cavernoma of patient II-2: T2 weighted sequence shows circumscribed lesion with a typical “popcorn” or “berry” texture in the upper portion of the vermis and the superior left cerebellar peduncle (white arrow) establishing the diagnosis of cerebral cavernoma. An additional digital subtraction angiography did not display any other vascular malformation.

Autosomal dominant polycystic kidney disease (ADPKD) is a common hereditary systemic disorder caused by mutations in either *PKD1* or *PKD2*. It is characterized by the development of renal cysts and various extrarenal cystic and non-cystic manifestations (1). The cystic extrarenal disease occurs in other organs, such as liver, pancreas, seminal vesicles, and meninges. Non-cystic manifestations include intracranial aneurysms and dolichoectasias, aortic root dilatation and aneurysms, mitral valve prolapse and abdominal wall hernias (2). Cerebral cavernous malformation (CCM) is a vascular malformation characterized by closely clustered and enlarged vessels lined by a single layer of endothelium without intervening brain parenchyma (3). Development of CCM is linked to the presence of mutations within *KRIT1* (*CCM1*), *CCM2*, and *PDCD10* (*CCM3*). CCMs can cause variable neurological manifestations such as seizures, headaches, progressive or transient focal neurologic deficits, and cerebral hemorrhages. Recently, a patient with both ADPKD and CCM was reported, although his family history did not reveal any ancestor affected by this vascular malformation (4). Here, we report on a family with a history of ADPKD that is associated with CCMs (Figure 1).

## Patient case

A 49-year-old woman (patient II-2 in Figure 1) with ADPKD (multiple bilateral cysts, estimated glomerular filtration rate 87 ml/min) presented with a 2-day history of a rotary vertigo of sudden onset. The vertigo was triggered by specific head positions and on clinical examination a positive Dix-Hallpike maneuver could be detected. The remaining neurological examination was unremarkable. The patient was initially assigned to the clinic of otolaryngology with the suspected diagnosis of benign paroxysmal positional vertigo (BPPV). After 2 days, she was discovered to have an irregular upbeat nystagmus that was aggravated under visual-fixation suppression with Frenzel goggles, and a slight ataxia of the left arm. An MRI scan showed a circumscribed lesion in the upper portion of the vermis and the superior left cerebellar peduncle (Figure 1, right panel). The lesion appeared with typical “popcorn” or “berry” texture and a rim of signal loss in the fluid-attenuated inversion recovery (FLAIR) sequence. Susceptibility weighted imaging (SWI) revealed hemosiderin deposition within the rim (not shown). A surrounding edema suggested a recent bleeding event. Additional digital subtraction angiography (DSA) did not reveal any other vascular malformation or aneurysm (Figure 1, right panel). Despite having aortic isthmus stenosis, the patient had no other clinical data of interest. The patient was referred to our intermediate care station and after 2 days the upbeat nystagmus as well as vertigo and nausea had declined. A conservative approach was chosen to treat the vascular lesion. Her family history was limited, but she knew that her father (I-1) was affected by ADPKD and that both of her sisters (II-1 and II-3) have kidney cysts as well as her older sister's son (III-3). These data established the clinical diagnosis of ADPKD (Figure 1). Two years previously, her older sister (II-3) was discovered to have a CCM in the brain stem. This finding suggests a familial association of ADPKD and CCM. Mutation analysis by next-generation sequencing of the index patient (II-2) demonstrated a novel frameshift mutation c.12230_12239 del (p.Ala4077Glyfs^*^118) in exon 45 of the *PKD1* gene leading to a premature stop codon which has not been previously reported. Sequencing of *PKD2, KRIT1* (*CCM1*), *CCM2*, or *PDCD10* (*CCM3*) did not reveal any additional mutations.

## Discussion

ADPKD is the most common inherited renal cystic diseases, a group of disorders with related but distinct pathogenesis, characterized by the development of renal cysts and various extrarenal manifestations (1). It has been long known that extrarenal manifestations include intracranial aneurysms. The occurrence of intracranial bleeding in patients with ADPKD is most commonly caused by hypertensive intracerebral hemorrhage and less frequently by the rupture of intracranial aneurysm. However, also rare cases of arteriovenous malformation and pontine angioma associated with ADPKD have been reported (5, 6). The association of ADPKD with CCM and aneurysms has been recently described in one patient, but other family members did not show any vascular manifestations of ADPKD (4). Here, we report the occurrence of CCM in two sisters of a family affected by ADPKD caused by a novel *PKD1* mutation.

ADPKD is a genetically heterogeneous disease that is most frequently transmitted by an autosomal dominant pattern of inheritance involving mutations in one of two different genes: *PKD1* encoding for polycystin-1 (PC1) accounts for 85% of cases and *PKD2* encoding for polycystin-2 (PC2) accounts for approximately 15% of cases (1). While patients with *PKD2* mutations usually have a mild course of disease, individuals with *PKD1* mutations have a more severe disease phenotype (1). Especially mutations in the 5' region of *PDK1* have been associated with a worse renal phenotype when compared to mutations in the 3' region (18.9% vs. 39.7% with adequate renal function at the age of 60 years) and patients with 5' mutations tend to have a higher frequency of vascular complications such as intracranial aneurysms and aneurysm ruptures (7, 8). Although the novel frameshift mutation in the presented case is located within the 3' region of *PKD1* (exon 45) and thus less likely to be associated with a vascular phenotype according to these studies, Rossetti et al. have also mentioned 4 patients with mutations in exon 45 that had severe vascular complications (8). Physiologically, PC2 is a non-selective cation channel that is highly permeable to Ca^2+^ and is regulated by direct molecular interaction with the C-terminal tail of PC1 forming a ‘receptor-ion channel complex' (9, 10). In endothelial cells, luminal sheer stress leads to activation of PC1 which in turn allows calcium influx through the calcium-permeable channel PC2 (11, 12).

CCM is a vascular malformation characterized by closely clustered capillary-like channels. Clinical manifestations include seizures, hemorrhagic stroke and focal neurological deficits (13). Both sporadic and familial forms of CCM exist. Familial CCM is associated with a heterozygous germline loss-of-function mutation in *KRIT1* (*CCM1*), *CCM2* or *PDCD10* (*CCM3*) (13). Although loss of any CCM gene causes very similar disease phenotypes, CCM proteins are structurally distinct and share no sequence homology. Functionally, CCM proteins were shown to regulate diverse aspects of endothelial cell morphogenesis and blood vessel stability, such as cell–cell junctions, cell shape and polarity, or cell adhesion to the extracellular matrix (14). CCM1 and CCM2 form a protein complex and their physical interaction is necessary for correct protein localization at endothelial cell-cell junctions and stabilization of vascular integrity (15). Interestingly, PC1 also plays a pivotal role in the structural integrity of blood vessels and cell-cell interactions (16, 17). As PC1 and PC2 as well as CCM1-3 are involved in sustaining vascular integrity, and the pathogenesis of both ADPKD and CCM follows the *two-hit* model with biallelic mutations including one germline and one somatic mutation, it is tempting to speculate that specific *PDK1* germline mutations might represent a predisposition for the development of CCM. Further studies are necessary to elucidate the role of PC1 and PC2 in vascular integrity.

We report the occurrence of CCMs in two patients with polycystic kidney disease that harbor a novel *PKD1* frameshift mutation and lack *CCM1-3* mutations. Thus, as in the case of aneurysmal subarachnoid hemorrhage (18), some ADPKD patients might be at risk for intracerebral hemorrhage caused by CCMs at a younger age. We suggest that CCM might be another manifestation of the systemic vasculopathy in ADPKD, although we cannot exclude the possibility of a simultaneous mutation in a hitherto unknown gene that is associated with CCM. Further research is warranted to determine the rate of CCM among ADPKD patients and the prevalence of PKD among patients with CCM or cryptogenic hemorrhage.

## Ethics statement

No investigations or interventions were performed outside routine clinical care for this patient. As this is a case report, without experimental intervention into routine care, no formal research ethics approval was required; Written, fully informed consent was given and recorded from the patient.

## Author contributions

CT acquisition of data, analysis and interpretation of data, writing of the manuscript; AZ acquisition of data, critical revision of manuscript for intellectual content; KH acquisition and interpretation of data; LM acquisition and interpretation of data; OG acquisition of data; PK critical revision of manuscript for intellectual content; HW critical revision of manuscript for intellectual content; SM study concept and design, critical revision of manuscript for intellectual content.

### Conflict of interest statement

KH and LM were employed by company CeGaT GmbH. The other authors declare that the research was conducted in the absence of any commercial or financial relationships that could be construed as a potential conflict of interest.
